# D-Serine metabolism in C6 glioma cells: Involvement of alanine-serine-cysteine transporter (ASCT2) and serine racemase (SRR) but not D-amino acid oxidase (DAO)

**DOI:** 10.1002/jnr.22332

**Published:** 2010-06

**Authors:** Pilleriin Sikka, Rosie Walker, Rebecca Cockayne, Matthew JA Wood, Paul J Harrison, Philip WJ Burnet

**Affiliations:** 1 Department of Psychiatry, University of Oxford, Warneford HospitalOxford, United Kingdom; 2 Department of Physiology, Anatomy and Genetics, University of OxfordOxford, United Kingdom

**Keywords:** D-serine, eliminase, racemase, uptake, transporter, glia, DAAO

## Abstract

D-serine is an endogenous N-methyl-D-aspartate (NMDA) receptor coagonist. It is synthesized from L-serine by serine racemase (SRR), but many aspects of its metabolism remain unclear, especially in the forebrain, which lacks active D-amino acid oxidase (DAO), the major D-serine degradative enzyme. Candidate mechanisms include SRR operating in α,β-eliminase mode (converting D-serine to pyruvate) and regulation by serine transport, in which the alanine-serine-cysteine transporter ASCT2 is implicated. Here we report studies in C6 glioma cells, which “simulate” the forebrain, in that the cells express SRR and ASCT2 but lack DAO activity. We measured D-serine, ASCT2, SRR, and DAO expression and DAO activity in two situations: after incubation of cells for 48 hr with serine isomers and after increased or decreased SRR expression by transfection and RNA interference, respectively. Incubation with serine enantiomers decreased [^3^H]D-serine uptake and ASCT2 mRNA and increased SRR immunoreactivity but did not alter DAO immunoreactivity, and DAO activity remained undetectable. SRR overexpression increased D-serine and pyruvate and decreased [^3^H]D-serine uptake and ASCT2 mRNA but did not affect DAO. SRR knockdown did not alter any of the parameters. Our data suggest that D-serine transport mediated by ASCT2 contributes prominently to D-serine homeostasis when DAO activity is absent. The factors regulating D-serine are important for understanding normal NMDA receptor function and because D-serine, along with DAO and SRR, is implicated in the pathogenesis and treatment of schizophrenia. © 2010 Wiley-Liss, Inc.

The neutral amino acid D-serine acts on the glycine binding site of the N-methyl-D-aspartate receptor (NMDAR) and modulates glutamate-mediated receptor activation. The importance of D-serine in mammalian brain function is apparent from extensive investigations (for reviews see Snyder and Kim,^2000^; Martineau et al.,^2006^), including roles in synaptic plasticity (Zhang et al.,^2008^) and memory (Duffy et al.,^2008^). Furthermore, D-serine is implicated in the pathophysiology and therapy of several psychiatric and neurological conditions (Martineau et al.,^2006^). In schizophrenia, there is evidence that D-serine levels are decreased (Hashimoto et al.,^2005^; Bendikov et al.,^2007^), a deficiency that may contribute to the proposed NMDAR hypofunction of the disorder (Olney and Farber,^1995^; Coyle et al.,^2003^) and that has led to D-serine replenishment as a novel therapeutic strategy (Coyle and Tsai,^2004^; Tuominen et al.,^2005^).

D-serine is synthesized from L-serine by serine racemase (SRR), and it is degraded by D-amino acid oxidase (DAO). In the brain, both SRR and DAO are primarily but not exclusively localized to glial cells (Wolosker et al.,^1999^; Mustafa et al.,^2004^; Kartvelishvily et al.,^2006^; Miya et al.,^2008^). D-serine uptake into glial cells is mediated largely by ASCT2 (Hayashi et al.,^1997^; Shao et al.,^2009^). Beyond the basic details, however, much remains unclear about how D-serine levels are regulated. A specific complexity pertains in the cerebral cortex, wherein D-serine and SRR are abundant (Schell et al.,^1995^,^1997^; Wolosker et al.,^1999^), and, although DAO is expressed (Bendikov et al.,^2007^; Verrall et al.,^2007^), its activity is minimal (Horiike et al.,^1994^; Wang and Zhu,^2003^). There are two possibilities for cortical D-serine regulation in the virtual absence of DAO. One is that SRR can remove D-serine via an α,β-eliminase reaction (Foltyn et al.,^2005^) that occurs in addition to, rather than in lieu of, its racemase activity (Panizzutti et al.,^2001^; Strísovský et al.,^2003^,^2005^; Nagayoshi et al.,^2009^). SRR may also operate in reverse racemase mode, converting D- to L-serine (Foltyn et al.,^2005^). This multifunctional capability of SRR could therefore be sufficient to regulate D-serine in the forebrain and thus, preclude the need for active DAO. A second possible mechanism of D-serine regulation may be via transport and recycling between cells and synapses (and between periphery and brain) rather than via degradation. Given that ASCT2 modulates the levels of its high-affinity substrate glutamine (Dolińska et al.,^2001^), it is reasonable to propose that it might also regulate intracellular D-serine homeostasis.

Given the major role of glia in D-serine metabolism, glial cells provide a useful in vitro model system with which to investigate these issues (Shoji et al.,2006a; Martineau et al.,^2008^). Moreover, the rat C6 glioma cells lack functional DAO despite expressing it (Park et al.,^2006^; Burnet et al.,^2009^). These cells are useful for studying D-serine in a cellular environment that lacks DAO, because in this respect they “simulate” the situation in the forebrain. Here we have studied C6 cells to address two main questions. 1) Which components of serine metabolism (SRR, DAO, D-serine uptake, and ASCT2) respond to changes in serine concentrations in glial cells that, at least under basal conditions, have no active DAO? We have measured SRR and DAO expression and activity, [^3^H]D-serine uptake, and ASCT2 mRNA expression in C6 glioma cells 48 hr after the addition of serine isomers. 2) What influence does increased and decreased expression of SRR, achieved by transfection and RNA interference, respectively, have on the same parameters? Some of these data have been presented in abstract form (Burnet et al.,^2009^).

## MATERIALS AND METHODS

### Materials

2,2′-Azino-bis(3-ethylbenzthiazoline-6-sulphonic acid; ABTS), Bradford reagent, Dulbecco's modified Eagle's medium (DMEM), glutaraldehyde, L-glutamine, fetal calf serum (FCS), L- and D-serine, D-proline, 0.25% tripsin/EDTA solution, Kodak BioMax X-Omat AR film, RIPA buffer, and Tri reagent were purchased from Sigma-Aldrich (Poole, United Kingdom). The “Kaleidescope” protein molecular weight marker and the chemiluminescence ECL-Plus kit were purchased from GE Healthcare (Buckinghamshire, United Kingdom). Polyclonal anti-mouse SRR and anti-D-serine were purchased from Bioline and Abcam (Cambridge, United Kingdom), respectively. Polyclonal anti-human DAO was a generous gift from Dr James Kew (GSK, Harlow, United Kingdom), and anti-rabbit and anti-mouse peroxidise-linked (HRP-linked) antisera were purchased from Bio-Rad. Polyvinyl difluoride (PVDF) membranes were purchased from Immobilon-P (Millipore, Watford, United Kingdom). [^3^H]D-serine (25 Ci/mmol) was purchased from Perkin Elmer (Norwalk, CT). The pyruvate assay was purchased from BioVision United Kingdom. Turbofect was purchased from Fermentas. The pcDNA3.1 and pcDNAEGFP plasmids and Amplex red were purchased from Invitrogen.

### Cell Culture

Rat glioma C6 cells (from the European collection of Animal Cell Cultures; ECACC) were cultured in Dulbecco's modified Eagle's medium containing 10% (v/v) FCS and supplemented with 2 mM L-glutamine, at 37°C in a humidified atmosphere of 5% CO_2_. For all experiments, C6 cells were seeded into six-well plates (2 × 10^5^ cells in 2 ml growth medium in each well) and allowed to adhere for 24 hr. The culture medium was then replaced with either fresh medium containing plasmid lipid complexes (see below) or L- or D-serine or D-proline at a final concentration of 2 or 20 mM, which are similar to the amounts of D-amino acids used in other studies of C6 cells (Park et al.,^2006^). D-proline is not transported into C6 cells and therefore was chosen as a control for extracellular hypertonicity, which occurs when amino acids are added to the culture medium. In all studies, cells were incubated for 48 hr following pharmacological or molecular intervention.

### Western Blotting

C6 cells were harvested with 0.25% trypsin/EDTA solution, transferred to a 1.5-ml microfuge tube, and centrifuged for 1 min at 12,000*g*. The cell pellet from two wells of the control and experimental condition was solubilized in 40 μl RIPA buffer and used for Western blotting as previously described (Verrall et al.,^2007^). The remaining cells were used for RNA extraction (see below). Briefly, 16 μg protein from each sample was loaded into separate wells of a 12% SDS/polyacrylamide gel and fractionated by electrophoresis along side a “Kaleidascope” molecular weight marker. Pilot studies established that 16 μg protein allowed the detection of SRR, DAO, and β-actin immunoreactivities within the linear range. Fractionated samples were then transblotted onto PVDF membranes.

The membranes were blocked with 2% (w/v) nonfat milk in PBS containing 0.1% Tween 20 (PBST) for 1 hr and then incubated with primary antibody (anti-mouse SRR, 1:1,000; anti-human DAO, 1:1,000) in PBST containing 1% (w/v) BSA for 1.5 hr at room temperature. Membranes were then washed three times for 10 min each in PBST and incubated for 1 hr in HRP-linked anti-mouse or anti-rabbit antibody in blocking buffer. Immunoreactive bands were visualized by chemiluminescence using the ECL-Plus kit and apposing membranes to X-ray film.

### RT-PCR

Total RNA was extracted from cells using the Tri reagent method. Reverse transcription of 2 μg RNA and PCR were performed as previously described (Burnet et al.,^2008^), using primers against rat ASCT2 (forward 1250–1272 bp; reverse 1481–1500 bp; accession No. NM_175758), SRR (forward 841–863 bp; reverse 1082–1101 bp; accession No. NM_198757), DAO (forward 571–593 bp; reverse 801–822 bp; accession No. NM_053626), or GAPDH (forward 11–34 bp; reverse 251–273 bp; accession No. NM_017008). Amplification parameters for each gene were 94°C, 30 sec; 62°C, 30 sec; 72°C, 1 min; for up to 35 cycles. Amplimers were fractionated on a 2% agarose gel in Tris-acetate-EDTA (TAE) buffer, pH 7.4, containing 5 μg/ml ethidium bromide. Bands were visualized with UV illumination using an Alpha Imager 3400. Nonsaturating levels of each amplimer were predetermined by removing a sample of the reaction mix during amplification after several cycles (25–35) and visualizing them on a gel. The amplimer intensities were plotted against cycle number to determine the cycles required to achieve about 50% intensity (amplification) of each gene. For quantitative analysis, the abundance of ASCT2 mRNA was expressed as ratio of ASCT2/GAPDH intensities.

### D-Serine Uptake

The uptake of D-serine into C6 cells was performed according to the method described by Hayashi et al. (1997). Briefly, adhered cells were preincubated with 1 ml uptake buffer (5 mM HEPES, 5 mM KCl, 1 mM KH_2_PO_4_, 140 mM NaCl, 1.8 mM CaCl_2_, 0.4 mM MgSO_4_, 5 mM D-glucose, adjusted to pH 7.4 with NaOH) for 10 min at 37°C. The uptake reaction was initiated by the addition of 0.2 ml of the uptake buffer containing [^3^H]D-serine; for saturation experiments, a range of [^3^H]D-serine concentrations (1–8 mM) was used. Nonspecific uptake of [^3^H]D-serine was estimated by performing the same experiments on ice (0–4°C). After incubation for 10 min at 37°C, the reaction was stopped by washing cells three times in 1 ml ice-cold uptake buffer. Cells were then solubilized with 0.1 M NaOH (500 μl), of which 400 μl was used to determine accumulated radioactivity by liquid scintillation spectroscopy and the remainder to measure protein concentrations in each sample. Specific [^3^H]D-serine uptake was defined as the difference between values obtained at 37°C vs. 0–4°C. The uptake of [^3^H]D-serine into manipulated or amino-acid-treated C6 cells was determined at the Km. This was calculated from an Eadie-Hofstee plot of the saturation data (Hayashi et al.,^1997^), where the Km was the reciprocal of the slope of the graph.

### D-Serine and Pyruvate Measurements

The measurement of D-serine and pyruvate concentrations were used as indices of SRR racemase and α,β-eliminase activities, respectively. The D-serine enzyme-linked immunosorbant assay (ELISA) was performed by using a modification of a recently reported protocol (Zhang et al.,^2008^). Cells were detached from wells with 0.25% trypsin/EDTA solution and pelleted in 1.5-ml Eppendorf tubes. Cell pellets were then homogenized in ice-cold distilled water, and debris was removed by centrifugation. The concentration (mg/ml) of soluble protein in each sample was determined by using the Bradford reagent for colorimetric measurements at 595 nm, with BSA standards. Protein in each extract was adjusted to 0.5 mg/ml with water. Twenty-five microlitres of glutaraldehyde was added to 100 μl of each extract or D-serine standards (0.06–15 nmol/ml) in PBS containing 0.2 mg/ml BSA (resulting in a final concentration range of D-serine standards of 0.02–5 nmol). The solutions were mixed thoroughly, and 50-μl triplicates of this were added to an ELISA plate. After a 2-hr incubation at room temperature, wells were washed with PBST and air dried. Blocking buffer (2% nonfat milk in PBST, 50 μl) was added to the wells and incubated for 30 min at room temperature. Plates were then washed three times in PBST (300 μl/well). The D-serine specific antibody was diluted 1:1,000 in PBST containing 1% BSA. Fifty microliters was added to each well and incubated for 1 hr, followed by three washes with PBST. The HRP-linked anti-rabbit antibody was then added for 30 min, followed by three washes in PBST. A colorimetric reaction initiated by the addition of 50 μl ABTS to each well was measured at 405 nm.

In separate experiments, cell pellets (see above) were resuspended in 50 μl water and then boiled for 5 min to denature endogenous lactate dehydrogenase in the samples (De Miranda et al.,^2002^). Preliminary experiments confirmed that boiling and did not degrade sodium pyruvate (not shown). The suspensions were then centrifuged to remove cell debris, and the supernatants were retained for analysis. The concentration of pyruvate in all samples and boiled sodium pyruvate standards (40–200 mM) was estimated in a pyruvate oxidase-based colorimetric assay (Biovision).

### DAO Activity Assays

DAO activity was measured via the estimation of hydrogen peroxide formation as previously described (Burnet et al.,^2008^). Briefly, control and experimental cells were harvested from cell culture plates as described above and centrifuged at 12,000*g* for 1 min. Cell pellets were then homogenized in cold sodium phosphate buffer (50 mM Na_2_HPO_4_, pH 8.0) on ice. Ten microliters of the homogenate was added to a solution of Amplex red (0.5 μg), horseradish peroxidise (10 μg), and D-proline (50 mM) in a total volume of 20 μl phosphate buffer. After 1 hr of incubation at 37°C, cell debris was pelleted, and the absorbance of the supernatant was measured at 571 nm. Baseline measurements were made in reaction solutions that did not contain D-proline. DAO activity in C6 cells transfected with recombinant rat DAO (rDAO), using similar methods, was also determined. Synthesis of the rDAO is described below.

### Overexpression and RNA Interference

For overexpression studies, the open reading frames of rat SRR (accession No. NM_198757) and rat DAO (accession No. NM_053626) were amplified and cloned into the pcDNA 3.1 vector using standard protocols. The pcDNAEGFP construct was used as a control plasmid. The C6 cells were transfected with SRR, DAO, or enhanced green fluorescent protein (EGFP) plasmids 16 hr after seeding into six-well plates, using TurboFect according to the manufacturer's recommendations. A total of 3 μg of DNA was added to each well, ensuring that for each plate three wells received pcDNA-EGFP (control transfection) and the other three wells received SRR or DAO (experimental transfection). Cells were then incubated for a further 48 hr, prior to protein extraction, DAO activity assays, D-serine uptake studies, and measurements of D-serine and pyruvate.

Plasmid constructs of U6-driven small hairpin RNAs (shRNAs) were purchased from Sigma-Genosys. Two of the shRNA plasmids targeting the rat SRR open reading frame (referred to as “sh596” and “sh886”) were transfected into C6 cells as described above, with pEGFP as the control in three wells of each six-well plate of cells. Cells were harvested 48 hr after transfection, and extracts were prepared for the measurements described for SRR overexpression.

### Data Analysis

All cell-culture data were analyzed by paired-samples *t*-test (SPSS version 15 for Windows), to compare control with experimentally manipulated cells in each plate.

## RESULTS

### Expression of DAO, SRR, and ASCT2 in C6 Cells

**Fig. 1:**
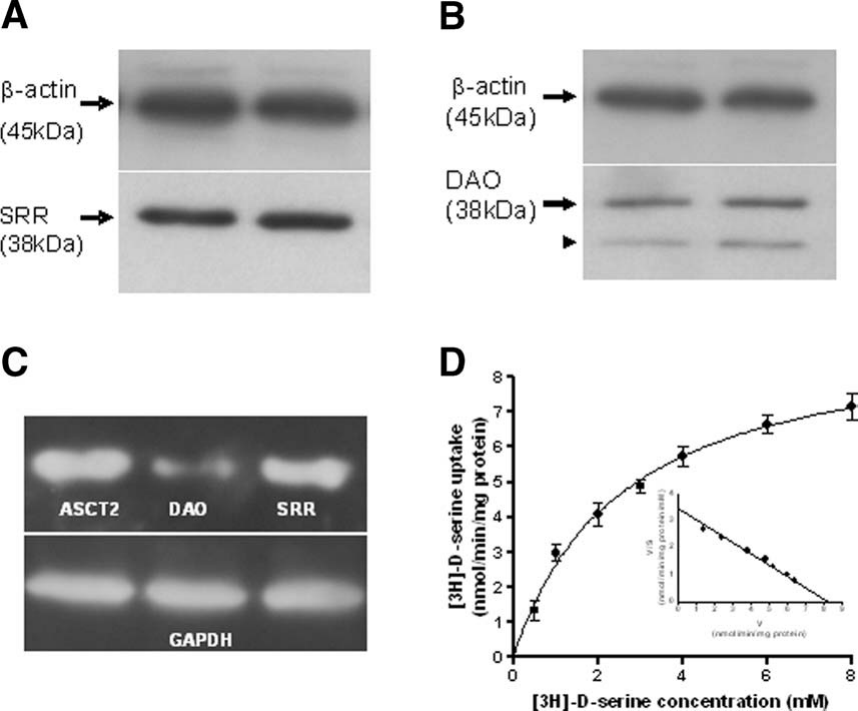
SRR, DAO, and ASCT2 expression and [^3^H]D-serine uptake in C6 glioma cells. **A:** Representative Western blots of SRR and β-actin in C6 cell extracts from two separate culture wells. **B:** DAO and β-actin Western blots of C6 cell extracts. Arrowhead indicates an additional minor band migrating at 30 kDa. **C:** Ethidium bromide-stained agarose gels demonstrating the relative abundance of ASCT2, DAO, SRR, and GAPDH mRNAs in C6 cells, determined by RT-PCR. **D:** Uptake of [^3^H]D-serine into C6 cells. Data points are mean ± SEM from four experiments. **Inset** shows an Eadie-Hofstee plot of these data for the calculation of Km.

Western blots of SRR and DAO immunoreactivity in C6 cells, relative to β-actin are shown in Figure 1A,B. Both enzyme proteins were expressed as a major band migrating at the predicted weights (∼38 kDa), with an additional minor DAO band at ∼30 kDa (Fig. 1B). The different affinities and dilutions of each antibody did not allow the direct comparison of the relative levels of each enzyme in protein extracts. However, the abundance of SRR mRNA was severalfold higher than that of DAO mRNA relative to GAPDH mRNA in RT-PCR experiments (Fig. 1C).

Concentration-dependent [^3^H]D-serine uptake was demonstrated in C6 cells (Fig. 1D). The affinity for [^3^H]D-serine transport into C6 cells (Km = 2.54 ± 0.15 mM) was similar to a published value of 2.48 ± 0.08 mM (Hayashi et al.,^1997^). Subsequent [^3^H]D-serine uptake experiments were performed at 2.5 mM. ASCT2 mRNA was readily detectable (Fig. 1C) and was similar to SRR mRNA abundance.

### Effect of Serine Isomers on SRR and DAO Immunoreactivity

**Fig. 2:**
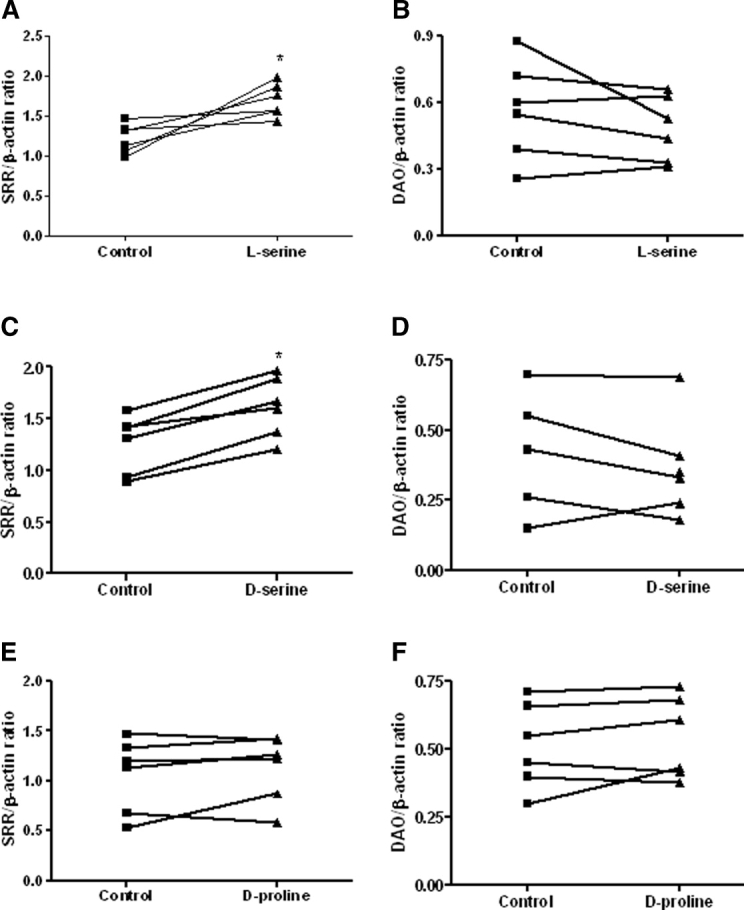
Effect of 20 mM serine isomers and D-proline on SRR and DAO immunoreactivity relative to β-actin in C6 cells. **A:** Increased SRR in cells after a 48-hr exposure to L-serine. **B:** No effect of L-serine on DAO expression. **C:** Increased SRR expression in cells after a 48-hr exposure to D-serine. **D:**No effect of D-serine on DAO. The administration of D-proline (20 mM) to cells did not alter SRR (**E**) or DAO (**F**). *P < 0.01

The addition of 20 mM L-serine (Fig. 2A) or D-serine (Fig. 2C) but not D-proline (Fig. 2E) to the culture medium increased the expression of SRR in C6 cells after 48 hr of incubation (L-serine: *t* = –5.44, *P* = 0.003; D-serine: *t* = –4.21, *P* = 0.008; D-proline: *t* = 0.43, *P* = 0.493). The lack of effect of D-proline demonstrated that the observed changes were not secondary to a hypertonic medium. None of the amino acids affected DAO immunoreactivity (Fig. 2B,D,F), and DAO activity remained undetectable (detection limit of assay in the absence of D-proline at 571 nm/mg protein/min = 0.02 ± 0.001), except after rDAO transfection, which was used as a positive control for the activity assay (571 nm/mg protein/min = 0.29 ± 0.02). The same effects on SRR immunoreactivity were observed with 1 mM of serine isomers, the magnitude of SRR increase being approximately one fifth of that observed with 20 mM (data not presented).

### Effect of Serine Isomers on [^3^H]D-Serine Uptake and ASCT2 mRNA

**Fig. 3:**
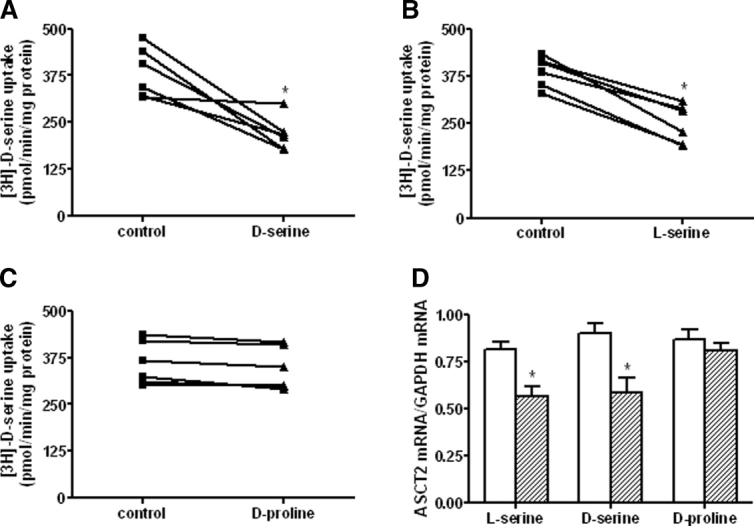
Effect of 48-hr exposure to 20 mM serine isomers and D-proline on [^3^H]D-serine uptake into C6 cells and ASCT2 expression. **A:** Reduced [^3^H]D-serine uptake after a 48-hr exposure to D-serine. **B:** Reduced [^3^H]D-serine uptake after exposure to L-serine. **C:** No effect of D-proline on [^3^H]D-serine uptake. **D:** L- and D-serine, but not D-proline, decreased ASCT2 mRNA (hatched bars) relative to controls (open bars). *P < 0.01, n = 6 for each condition.

Cells incubated for 48 hr with 20 mM D-serine (Fig. 3A) or L-serine (Fig. 3B) had significantly reduced uptake of [^3^H]D-serine compared with controls and cells incubated with D-proline (Fig. 3C; D-serine: *t* = 4.364, *P* = 0.007; L-serine: *t* = 8.5, *P* < 0.001; D-proline: *t* = –0.547. *P* = 0.591). RT-PCR demonstrated reduced ASCT2 mRNA in C6 cells treated with L- or D-serine compared with controls, paralleling the reduced D-serine uptake in the same cells (Fig. 3D; L-serine: *t* = 5.214, *P* = 0.003; D-serine: *t* = 5.619, *P* = 0.002; D-proline: *t* = 1.549, *P* = 0.182).

### D-Serine and Pyruvate Concentrations After Serine Incubation

**Fig. 4:**
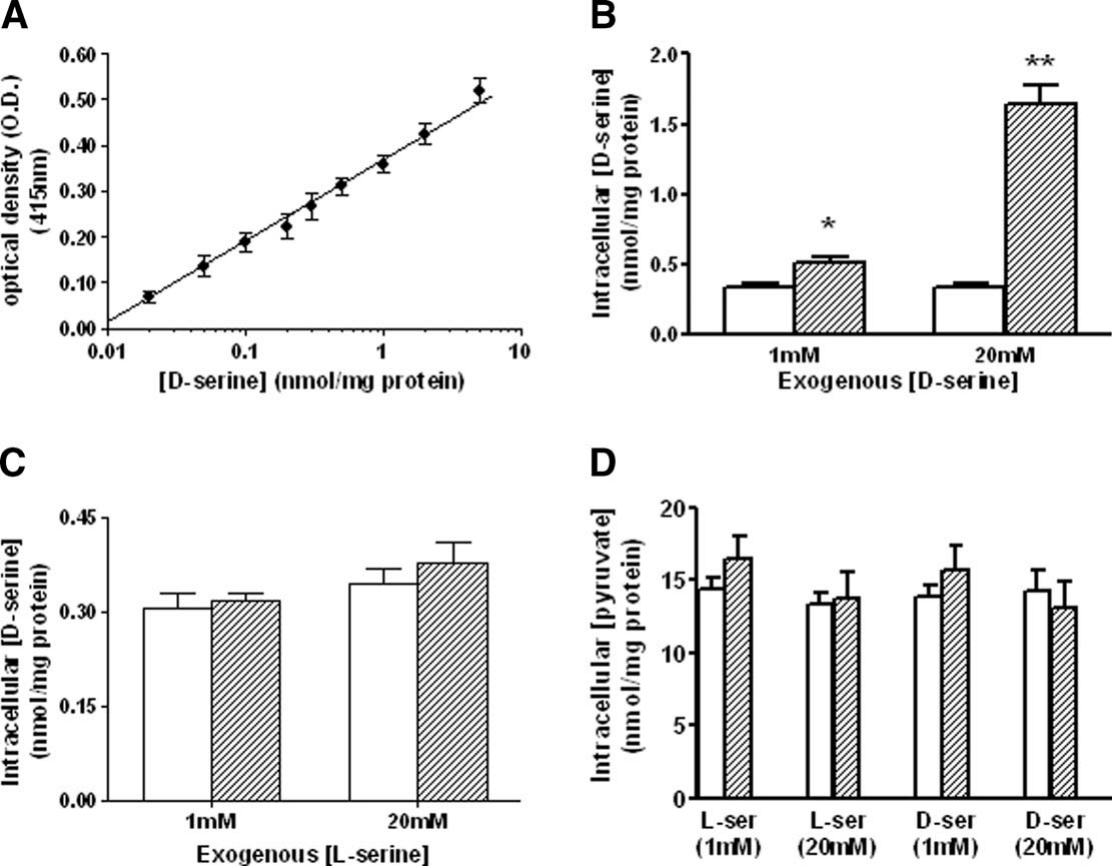
Measures of D-serine and pyruvate concentrations in C6 cells. **A:** Representative standard curve of D-serine ELISA generated by immobilizing the amino acid at several concentrations onto assay plates. Each data point represents the mean ± SEM from three experiments. **B:** Increased intracellular D-serine levels following a 48-hr incubation with D-serine in the culture medium (hatched bars) compared with controls (open bars). **C:** No effect of 48-hr incubation with L-serine on intracellular D-serine levels. **D:** Incubation with L-serine or D-serine (hatched bars) does not alter intracellular pyruvate concentrations compared with paired controls (open bars). *P < 0.05, **P < 0.001, n = 6 for each condition.

A representative ELISA standard curve using a solution of D-serine/BSA conjugate is shown in Figure 4A; the detection limit was approximately 0.01 nmol/mg protein of cell extract. The addition of 1 mM and 20 mM D-serine to C6 cells produced a half (*t* = –5.573, *P* = 0.003) and a fourfold (*t* = –10.747, *P* < 0.001) elevation in intracellular D-serine concentration, respectively, compared with controls (Fig. 4B). By contrast, the concentration of D-serine in cells incubated for 48 hr with 1 and 20 mM L-serine did not significantly differ from controls (Fig. 4C). The concentration of intracellular pyruvate was also unaltered after incubation with exogenous serine enantiomers (Fig. 4D).

### DAO, [^3^H]D-Serine Uptake, and ASCT2 mRNA Following SRR Overexpression and Knockdown

**Fig. 5:**
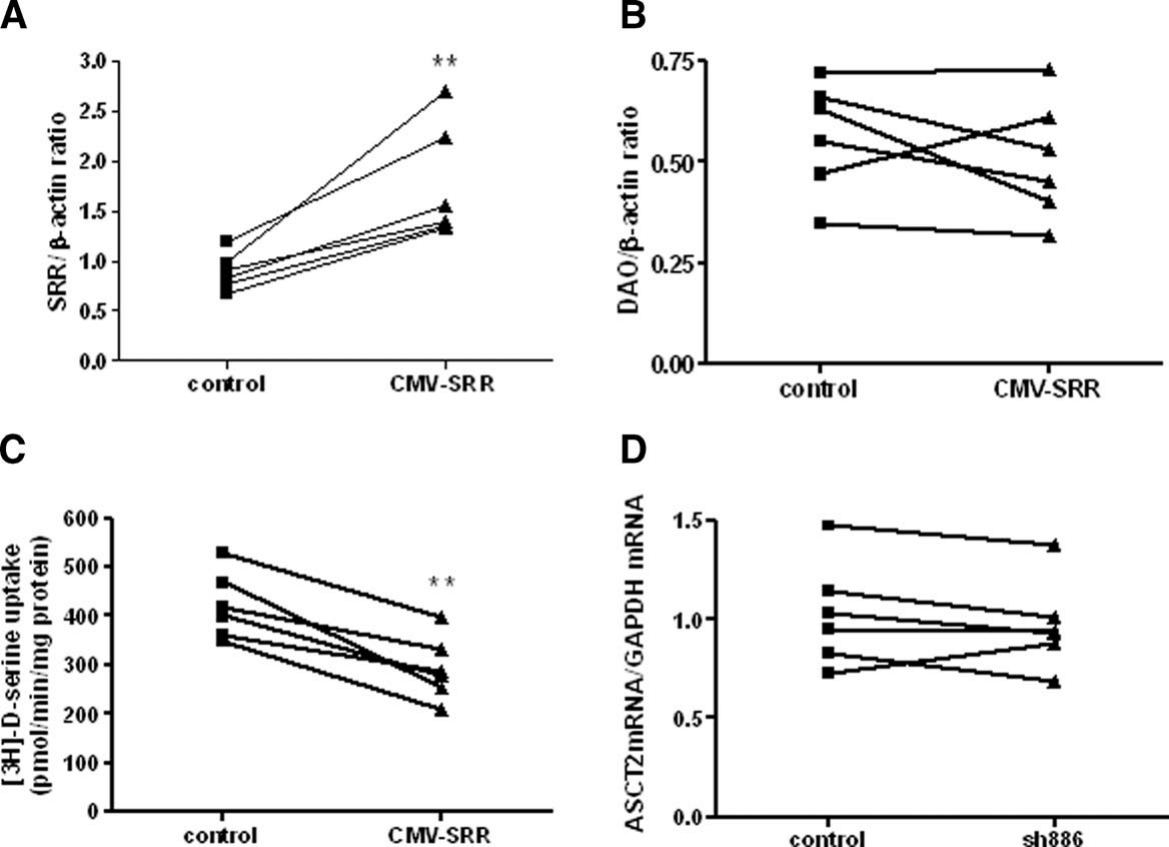
Effect of increased SRR expression in C6 cells on DAO and D-serine uptake and ASCT2 expression. **A:** Confirmation of SRR overexpression in C6 cells transfected with recombinant SRR (CMV-SRR). **B:** DAO immunoreactivity is not altered in cells overexpressing SRR. **C:** [^3^H]D-serine uptake into C6 cells is reduced in cells overexpressing SRR. **D:** ASCT2 mRNA is decreased in cells overexpressing SRR. **P < 0.01.

Results of the expression of recombinant rat SRR (CMV-SRR) in C6 cells is shown in Figure 5A. An overall twofold increase in SRR immunoreactivity was achieved (*t* = –4.196, *P* = 0.009). This did not affect DAO immunoreactivity (*t* = 1.79, *P* = 0.133; Fig. 5B) or DAO activity (remaining undetectable) but did decrease D-serine uptake (*t* = 6.22, *P* = 0.002; Fig. 5C) and ASCT2 mRNA expression (*t* = 3.26, *P* = 0.022; Fig. 5D) compared with controls. The intracellular concentrations of D-serine and pyruvate were also increased 48 hr after transfection with CMV-SRR (Table I).

**TABLE I:** D-Serine and Pyruvate Concentrations in C6 Cells 48 Hours After CMV-SRR Transfection or SRR RNAi (Mean ± SEM)

	[D-serine] (nmol/mg protein)	[Pyruvate] (nmol/mg protein)
Control	0.37 ± 0.02	15.17 ± 0.34
CMV-SRR	0.57 ± 0.07*	22.52 ± 1.2*
Control RNAi	0.38 ± 0.03	13.80 ± 1.55
SRR RNAi	0.36 ± 0.03	10.74 ± 0.72

* *P* < 0.01, paired-samples *t*-test (n = 6 for each condition).

**Fig. 6:**
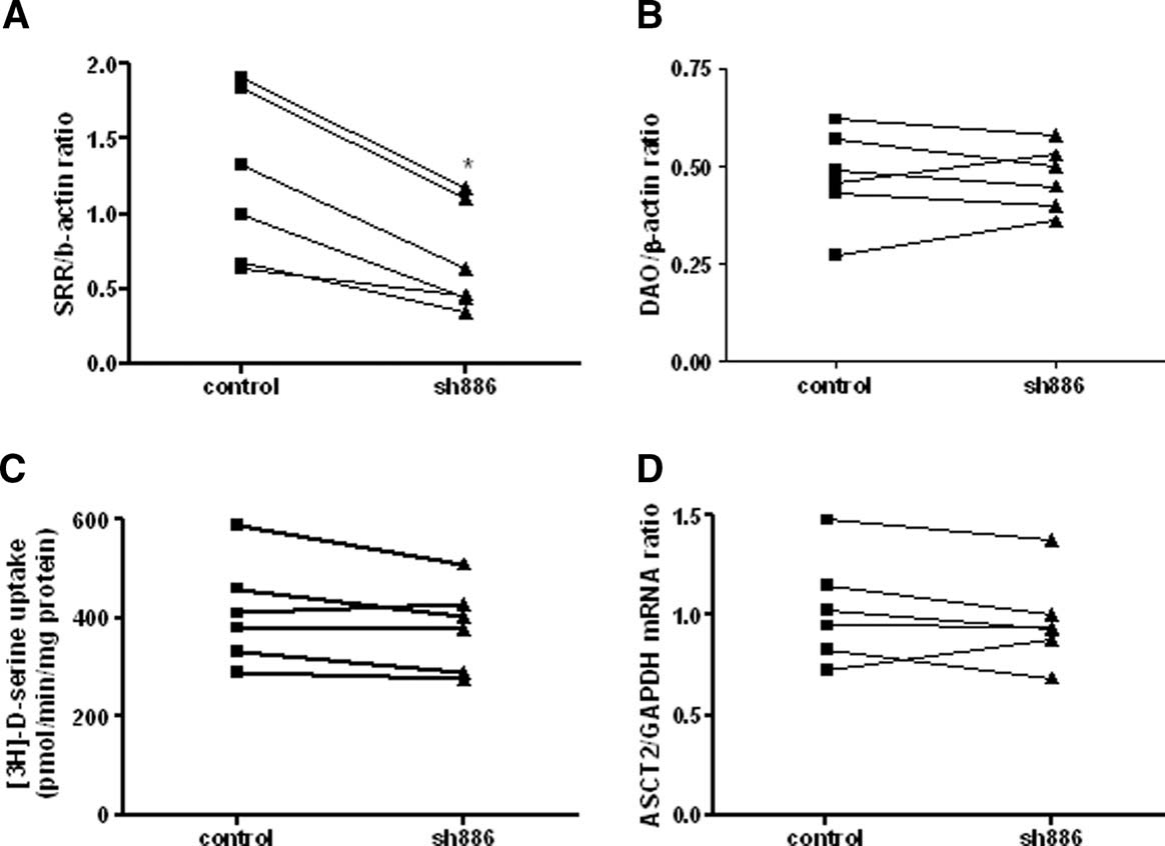
Effect of SRR RNAi in C6 cells on DAO and D-serine uptake and ASCT2 expression at 48 hr. **A:** Confirmation of SRR knockdown by RNAi in C6 cells transfected with plasmid sh886 compared with controls containing transfection reagent alone. **B:** The reduced expression of SRR does not affect the expression of DAO protein. **C:** [^3^H]D-serine uptake. **D:** ASCT2 mRNA relative to controls. *P < 0.01.

SRR immunoreactivity was reduced by approximately half when C6 cells were incubated for 48 hr after transfection with plasmid sh886 (*t* = 4.691, *P* = 0.005; Fig. 6A). Transfection with plasmid sh596 did not elicit a decrease in SRR protein (not shown). Cell viability did not appear to be affected by shRNAs as determined through cell counting with trypan blue (data not shown). The levels of DAO, DAO activity, D-serine uptake, ASCT2 mRNA, D-serine, and pyruvate in C6 cells transfected with sh886 were similar to controls (Fig. 6B–D, Tables I, II).

**TABLE II:** Summary of the Effects of Serine Administration and Molecular Manipulations on Components of the Serine Metabolic Pathway*

Manipulation	SRR protein	DAO protein	[^3^H]D-serine uptake	ASCT2 mRNA	Intracellular [D-serine]	Intracellular [pyruvate]
Addition of D-serine	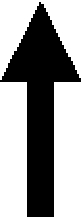	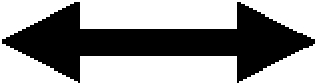	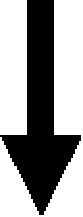	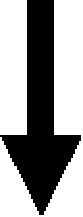	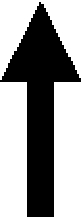	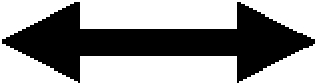
Addition of L-serine	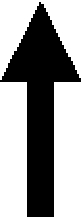	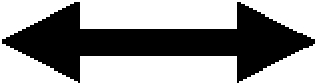	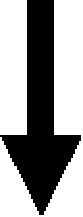	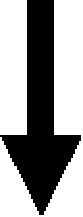	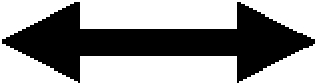	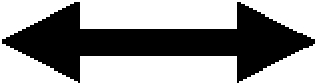
SRR overexpression	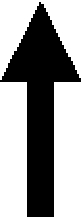	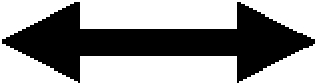	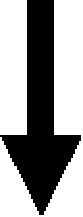	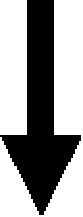	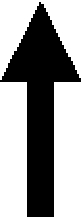	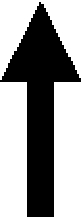
SRR knockdown	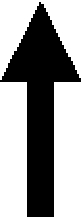	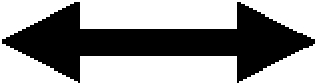	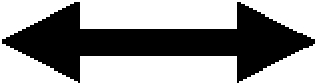	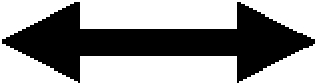	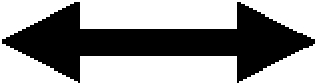	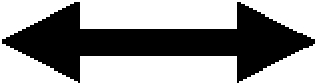

* Arrows indicate a significant increase 
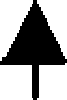
 or decrease or 
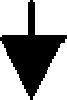
 no effect 
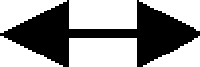
 for each manipulation on the parameters examined.

## DISCUSSION

The current study sought to explore the roles of SRR, DAO, and ASCT2 in the modulation of serine isomers in C6 glial cells, which contain undetectable DAO activity. A 48-hr time point was chosen so that the effects of SRR overexpression and SRR RNAi could also be evaluated. The results are summarized in Table II. The main findings are as follows. 1) DAO activity remains undetectable even if intracellular D-serine is increased by addition of D-serine or overexpression of SRR. 2) D-serine uptake and ASCT2 expression decrease after addition of either serine enantiomer and after SRR overexpression. 3) Addition of L-serine increased the abundance of SRR but did not lead to an increase in D-serine levels. 4) Addition of L- or D-serine did not alter pyruvate levels. 5) Knockdown of SRR had no effect on D-serine or its transport or pyruvate levels. 6) Transfection and overexpression of SRR led to increases in D-serine and pyruvate levels. These findings are briefly discussed in turn, before consideration of the potential significance for D-serine metabolism in glia in vivo.

### D-Serine Does Not Alter DAO Expression or Activity in C6 Cells

In glial cells of the hindbrain, DAO is abundantly expressed and active (Horiike et al.,^1994^) and is thought to underlie the very low levels of D-serine in the cerebellum (Schell et al.,^1997^). However, although DAO mRNA and immunoreactivity have been observed in glia (and neurons) of the forebrain (Moreno et al.,^1999^; Verrall et al.,^2007^), DAO activity in the cerebral cortex is minimal or undetectable. Similarly, although C6 cells express DAO (Fig. 1B), they also have no detectable DAO activity under basal conditions (Table I). Our data show that incubation of the cells with 20 mM L- or D-serine for 48 hr does not increase DAO (Fig. 2B,D), nor does DAO activity rise to measurable levels (Table I), nor was DAO or its activity altered by SRR overexpression (Fig. 5C) or knockdown (Fig. 6B). These results indicate that DAO is not a major player in D-serine metabolism in C6 cells and does not respond to changes in SRR expression, at least under the various conditions studied here. As in the forebrain, the significance of DAO being present but apparently inactive is unknown.

### Decreased [^3^H]D-Serine Uptake and ASCT2 mRNA After Incubation With Serine or SRR Overexpression

In the absence of functional DAO, we next investigated the possible roles of D-serine transport and expression of ASCT2 in the regulation of D-serine in C6 cells. We found support for this, in that D-serine uptake (Fig. 3A,B) and ASCT2 expression (Fig. 3D) both decrease after addition of either serine isomer. A similar but smaller effect with 1 mM serine (not shown), which is half the concentration of the Km for ASCT2, suggests some physiological relevance to the changes. D-serine uptake and ASCT2 mRNA also decrease after SRR overexpression (Fig. 5C,D). The fact that D-serine transport (Fig. 3C) and ASCT2 expression (Fig. 3D) were unaltered after incubation with D-proline indicates that the effects seen in response to L- or D-serine incubation are not a non-specific effect (e.g., of altered osmolarity). We conclude that reduced uptake is part of the homeostatic response of C6 cells to an increased extracellular concentration of serine isomers, serving to “close the flood gates” and prevent build up of nonphysiological concentrations of the amino acids inside the cell.

ASCT2 has been proposed to transport both serine enantiomers into C6 cells (Hayashi et al.,^1997^; Shao et al.,^2009^), so it is tempting to conclude that a decreased expression of ASCT2 contributes to the reduced transport seen after incubation with L- and D-serine, and indeed there was a significant correlation between [^3^H]D-serine uptake and ASCT2 mRNA (L-serine experiments: Pearson's R = 0.714, *P* = 0.009, n = 12; D-serine experiments: R = 0.730, *P* = 0.007, n = 12). However, it remains possible that other mechanisms, such as removal of ASCT2 from the membrane, and effects on other D-serine transporters are also involved in addition to regulation of ASCT2 expression.

### Relationships Between Serine and SRR in C6 Cells

Our final experiments focused on the response of SRR to changes in serine concentration and vice versa. The results were complex and not wholly as predicted by simple models of the relationship between an enzyme and its substrates and products.

Expression of SRR, in terms of its immunoreactivity, was increased 48 hr after incubation with L-serine, suggesting that its expression is responsive to its substrate concentration (Fig. 2A). SRR immunoreactivity was similarly enhanced by incubation with D-serine (Fig. 2B), a finding more difficult to interpret, given the various reactions that SRR can catalyze; that is, whereas D-serine is the product of its racemase activity, it is the substrate for the eliminase reaction and for the proposed reverse racemase reaction (Foltyn et al.,^2005^). In any event, despite the elevated SRR, neither racemase nor eliminase activities changed, in that D-serine did not rise after L-serine incubation (Fig. 4C) and pyruvate levels did not increase after either serine isomer (Fig. 4D). These results may imply that SRR is not active in C6 cells, at least under these conditions, a suggestion supported by the RNAi experiments, which showed that an ∼50% knockdown of SRR by RNAi (Fig. 6A) was also without effect on D-serine or pyruvate concentrations (Table II), DAO (Fig. 6B), D-serine transport (Fig. 6C), or ASCT2 mRNA (Fig. 6D). Another possibility is that SRR is active, accounting for the detectable basal levels of D-serine (Fig. 4B,C), but that the exogenously applied L-serine was converted to L-glycine by serine hydroxymethyl transferase (SHMT; Kohl et al.,^1980^), rather than to D-serine by SRR. On the other hand, if the L-serine did undergo SRR-mediated elimination, the resulting pyruvate might have been metabolized bythe Krebs cycle, thus precluding its detection. Parenthetically, SRR overexpression, induced by transfection, did lead to increased D-serine and pyruvate levels (Table II) in parallel with its increased abundance (Fig. 5A) and consistent with SRR acting in racemase and eliminase modes.

Overall, our results show evidence both for and against the role of SRR in the metabolism of D-serine in C6 cells and indicate the need for further studies. First, we must address the time course of the response to changing concentrations of serine isomers. For example, Shoji et al. (2006a) found that SRR activity was increased 16 hr after incubation of a human glioma cell line with D-serine, and it may be that a similar effect in C6 cells occurs but has subsided by the 48-hr time point studied here. Second, we must investigate further the relationship between SRR abundance and SRR activity. For example, it was recently shown that redistribution of SRR from cytosol to plasma membrane is a major determinant of SRR activity in glial cells (Mustafa et al.,^2009^) and in neurons (Balan et al.,^2009^). Any such intracellular relocation would not be detected by homogenate-based Western blots, so our immunoreactivity findings may not correspond to the abundance of active (as opposed to total) SRR. Also, there are many other factors known to regulate SRR activity, such as pyridoxal phosphate (De Miranda et al.,^2002^), nitrosylation (Shoji et al.,2006a,b; Mustafa et al.,^2007^), phosphotidylinositol (4,5)-bisphosphate (Mustafa et al.,^2009^), and ubiquitin-mediated degradation (Dumin et al.,^2006^), that have yet to be investigated in these cells.

### Extrapolation to the In Vivo Situation

The existing literature and the present findings show that the metabolism of D-serine and the roles played by SRR, DAO, and other key molecules is complex, and dependent in part on the nature and environment of the cells being studied. Clearly, this also constrains extrapolation to the situation pertaining in the brain in vivo. Nevertheless, two comments in this respect are worth making. First, our rationale for choosing C6 cells for this work was that they do not contain active DAO, even though they express the gene, a situation similar to that in the mammalian forebrain. It is therefore of interest that 14 days' administration of D-serine (50 mg/kg/day) increases SRR and decreases ASCT2 expression in rat frontal cortex (unpublished data), providing some support for the relevance of our findings to the in vivo situation. However it is not yet known whether this elevation of cortical D-serine affects endogenous SRR activity, and our in vitro findings emphasize the need for caution in making this assumption.

Finally, our results may have some relevance with regard to schizophrenia and its therapy. In that disorder, NMDAR hypofunction (Olney and Farber,^1995^; Coyle et al.,^2003^; Harrison and Weinberger,^2005^) is thought to occur, driven in part by co-agonist deficiency, especially of D-serine (Hashimoto et al.,^2005^; Bendikov et al.,^2007^). The cause of this is unknown, although both DAO and SRR have been identified as possible susceptibility genes (see Verrall et al.,^2009^). The findings have led to D-serine replenishment, DAO inhibition, and SRR enhancement being investigated as therapeutic strategies. Our results have three implications in this regard. First, they suggest that targeting of D-serine transport via ASCT2 may also be of value, comparable to the glycine transport inhibitors also being trialled in the disorder. Second, if D-serine levels are increased, via supplementation or via SRR enhancement, this will not be counteracted by induction of DAO activity. Third, the therapeutic value of DAO inhibition may be limited, in that its effect will likely be restricted to the cerebellum, with little or no effect in the forebrain (because DAO is inactive therein). In contrast, inhibition of D-serine transport might be expected to show a more widespread efficacy across brain regions.
